# A bioinformatics framework to identify the biomarkers and potential drugs for the treatment of colorectal cancer

**DOI:** 10.3389/fgene.2022.1017539

**Published:** 2022-09-27

**Authors:** Xiaogang Leng, Jianxiu Yang, Tie Liu, Chunbo Zhao, Zhongzheng Cao, Chengren Li, Junxi Sun, Sheng Zheng

**Affiliations:** Department of Colorectal and Anal Surgery, Weifang People’s Hospital, Weifang, China

**Keywords:** colorectal cancer, differential expressed gene, hub gene, survival analysis, *TIMP1*, drug repurposing

## Abstract

Colorectal cancer (CRC), a common malignant tumor, is one of the main causes of death in cancer patients in the world. Therefore, it is critical to understand the molecular mechanism of CRC and identify its diagnostic and prognostic biomarkers. The purpose of this study is to reveal the genes involved in the development of CRC and to predict drug candidates that may help treat CRC through bioinformatics analyses. Two independent CRC gene expression datasets including The Cancer Genome Atlas (TCGA) database and GSE104836 were used in this study. Differentially expressed genes (DEGs) were analyzed separately on the two datasets, and intersected for further analyses. 249 drug candidates for CRC were identified according to the intersected DEGs and the Crowd Extracted Expression of Differential Signatures (CREEDS) database. In addition, hub genes were analyzed using Cytoscape according to the DEGs, and survival analysis results showed that one of the hub genes, *TIMP1* was related to the prognosis of CRC patients. Thus, we further focused on drugs that could reverse the expression level of *TIMP1*. Eight potential drugs with documentary evidence and two new drugs that could reverse the expression of *TIMP1* were found among the 249 drugs. In conclusion, we successfully identified potential biomarkers for CRC and achieved drug repurposing using bioinformatics methods. Further exploration is needed to understand the molecular mechanisms of these identified genes and drugs/small molecules in the occurrence, development and treatment of CRC.

## Introduction

Colorectal cancer (CRC) is the most common subtype in gastrointestinal cancers, and its early symptoms are unobvious, which results in a high mortality rate. The continuous rise of new cases and deaths of CRC will lead to a significant increase in the economic burden globally (Rogler, 2014; Arnold et al., 2017; Hong et al., 2021; Liu et al., 2021). As the second leading cause of cancer death worldwide (Zhao et al., 2020; Sung et al., 2021), CRC has become a major global public health concern. Studies have shown that the clinical tumor stage at diagnosis affects the prognosis of patients. The 5-years relative survival rate of patients with stage I was 90%, while that of patients with stage IV was only 10% (Siegel et al., 2012; O'Connell et al., 2004; Yang et al., 2022). Currently, various diagnostic strategies for CRC include both invasive and non-invasive methods. Invasive methods rely on endoscopy and imaging. Imaging tests such as nuclear magnetic resonance (NMR) and computed tomography (CT) can be used to diagnose severe focal lesions, but both tests are expensive (Grassetto et al., 2012; Swiderska et al., 2014). Hence, there is an urgent need for alternative, cheap and easy-to-measure screening methods. Despite recent advances in treatment and multidisciplinary care, CRC patients continue to suffer from serious adverse reactions, which can impair prognosis and reduce survival (McQuade et al., 2017; Kong et al., 2020). The developing drugs with low toxicity, especially drug repositioning (Liu et al., 2020; Meng et al., 2022) is of great significance for improving the clinical treatment and reducing adverse reactions.

The improvement of molecular biology technology provides opportunities to develop more curative effect and enhance the outcomes of CRC. With the progress of high-throughput sequencing technology, gene expression profiling methods, such as RNA sequencing (RNA-seq), have been applied to scientific research and become a hot field of gene expression research (Saito et al., 2018; Deshiere et al., 2019; Zhang et al., 2021). The molecular mechanism of CRC holds the key to the prognosis and treatment response of patients, and is of great potential for the clinical practice (De Sousa et al., 2013; Sadanandam et al., 2013; Nguyen and Duong, 2018; Cheng et al., 2020; Cheng et al., 2021; Liu et al., 2022). Therefore, understanding of the molecular mechanism in the occurrence and development of CRC will help to develop novel therapies to optimize the treatment response throughout the disease course. In recent years, a large number of relevant CRC sequencing data have been generated, archived, and stored in public databases (Guo et al., 2017). Researches combining high-throughput sequencing data and bioinformatics analysis has gradually become a hot spot (Alves Martins et al., 2019; Zhao et al., 2019). Here, bioinformatics analysis of RNA-seq data of CRC patients may provide insights for drug repositioning for the treatment of CRC.

In this study, bioinformatics analysis was used to identify biomarkers of CRC and potential drugs that can improve the outcomes of CRC patients. Specifically, based on the TCGA data set and GSE104836 data set, we compared the transcriptome data of tumor samples and normal samples to identify differentially expressed genes (DEGs) on the two independent datasets. The DEGs were intersected for further analysis. Then these DEGs were further explored to detect the enriched GO terms and KEGG pathways. From those DEGs, latent drugs that can improve the prognosis of patients from the Crowd Extracted Expression of Differential Signatures (CREEDS) were also predicted. In addition, the hub genes in the protein-protein interaction (PPI) network were discovered according to the DEGs and survival analysis was carried out on these hub genes. Finally, drug candidates could reverse hub genes were also predicted by CREEDS and validated by literatures.

## Materials and methods

### Data collection

RNA-seq data of CRC patients were downloaded from the Cancer Genome Atlas (TCGA) database (https://portal.gdc.cancer.gov/) and the Gene Expression Omnibus (GEO) database (https://www.ncbi.nlm.nih.gov/geo/query/acc.cgi?acc=GSE104836). Meanwhile, the associated clinical information of 478 tumor samples and 41 normal samples from TCGA, and 10 patients and 10 healthy controls from the GSE104836 dataset was obtained.

### Differentially expressed gene analysis

DESeq2 is a R package that can identify DEGs from raw count data. It uses the contraction estimation of discrete and the fold change of the gene expression to improve the stability and interpretability of the estimation, which makes the more quantitative analysis focus on intensity (Love et al., 2014). DEGs in CRC tumor samples and normal samples were detected using DESeq2 package with the criteria of *p*-value < 0.001 and log2 |fold change| ≧ 2.

### Functional and pathway enrichment analysis of DEGs

After DEG analysis of the TCGA dataset and GSE104836 dataset, overlapping DEGs were screened, and then enrichment analysis of KEGG pathway and GO (The Gene Ontology, 2019) including biological process (BP), cellular component (CC), and molecular function (MF) were carried out to reveal the altered biological characteristics of CRC. The R packages “clusterProfiler” and “ggplot” were used to visualize the results of the enrichment analysis.

### PPI network and hub genes analysis

The online database STRING (http://string-db.org) was used to develop a PPI network of DEGs, and the minimum required interaction score was 0.7. The Cytoscape software was used to visualize the PPI network and to analyze the structural properties of the constructed network. The cytoHubba plug-in was used to identify hub genes in the PPI network.

### Potential drug identification

The CREEDS database consists of gene expression characteristics induced by single drug perturbation, which can be used to identify the relationship between genes, diseases, and drugs. To identify potential drugs for the treatment of CRC, we used the CREEDS database to find drugs that can reverse the DEGs. Specifically, for each drug in the CREEDS database, we calculated the *p*-value of the overlapping genes between downregulated genes of the drug and upregulated DEGs in CRC by hypergeometric test, and similarly, calculate the *p*-value of the overlapping genes between upregulated genes of the drug and downregulated DEGs in CRC. The drugs with any of the two *p*-value lower than 0.05 could be taken as candidates that could reverse the DEGs and might treat the CRC.

### Survival analysis

We obtained the OS time of all patients in TCGA database, and estimated the survival probability of CRC patients using Kaplan-Meier method. Kaplan-Meier survival curve was used to estimate the 50th percentile (median) of survival time and compare the survival distribution of two or more groups. Log-rank test was also used to compare the survival differences between groups. *p*-value <0.05 was considered to have significant differences between groups. The data were analyzed by R software.

## Results

### A framework of CRC related drugs repurposing

**FIGURE 1:**
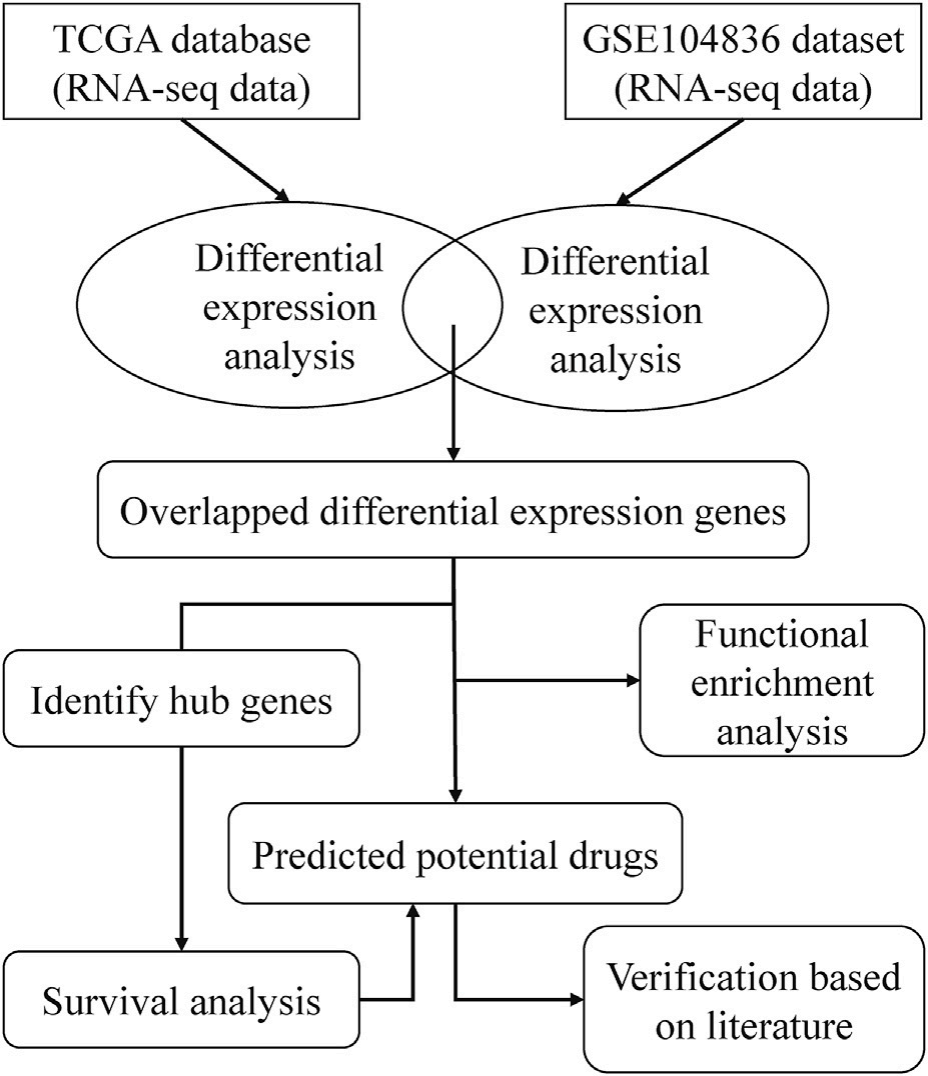
A brief workflow for drug repurposing.

To find drugs that can be used to treat CRC, we proposed a bioinformatics pipeline of drug repurposing based on transcriptome data. The workflow was shown in Figure 1 **.** After downloading the RNA-seq data from TCGA and GEO databases, we performed DEG analysis and pathway enrichment analysis. Then, the hub genes of DEGs were identified and survival analysis was done on the hub genes. According to the DEGs and CREEDS, drugs that could reverse the DEGs were identified, and 10 drugs can reverse the survival-related hub gene were further investigated. Finally, according to some previous studies, the effectiveness of the newly discovered drugs was verified.

### Patient characteristics

The RNA-seq data involved 478 tumor samples and 41 normal samples. There were 247 women and 272 men. 85 cases were at clinical stage I, 209 cases were at stage II, 140 cases were at stage III and 73 cases were at stage IV. Their average age was ∼67 years old. The clinical features of patients from the TCGA dataset were shown in Table 1 **.**

**TABLE 1:** General clinical information of CRC patients included in this study.

Characteristics	No	
Type		
	Tumor	478
	Normal	41
Average age	67.04	
Gender	Female	247
	Male	272
Tumor stage	Ⅰ	85
	Ⅱ	209
	Ⅲ	140
	Ⅳ	73
	Unknown	12

### DEGs identification

**FIGURE 2:**
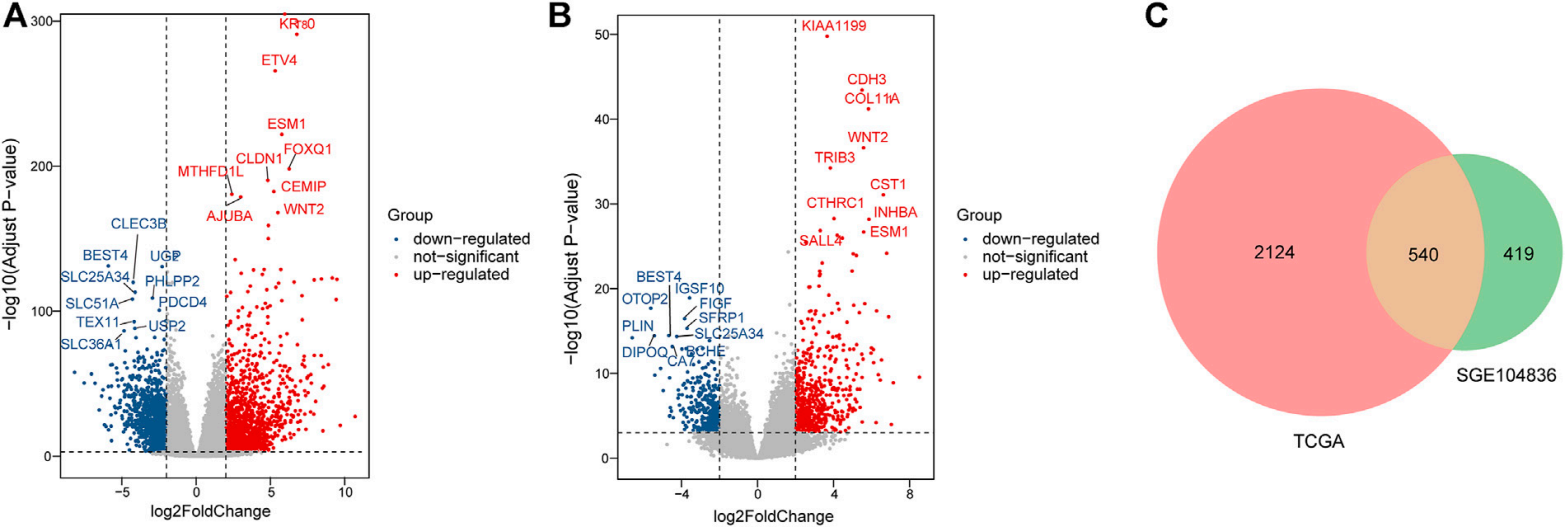
Identification of DEGs between tumor tissues and normal tissues in CRC patients. **(A–B)**. Differential expression volcanic map of **(A)**TCGA and **(B)** GEO dataset. Red dots indicate significant up-regulation, blue dots indicate significant down-regulation, and gray dots indicate genes with no significant changes. **(C)**. Venn plot for DEGs detected in two datasets.

In total, 2664 DEGs (1537 upregulated genes and 1127 downregulated genes) and 959 DEGs (563 upregulated genes and 396 downregulated genes) were extracted from TCGA (Figure 2A) and GSE104836 (Figure 2B) datasets respectively using p-value < 0.001 and llog2 |fold change| ≧ 2 as the cut-off criteria. A total of 540 DEGs (276 upregulated genes and 264 downregulated genes) were identified in both datasets (Figure 2C).

### Enrichment Analysis

**FIGURE 3:**
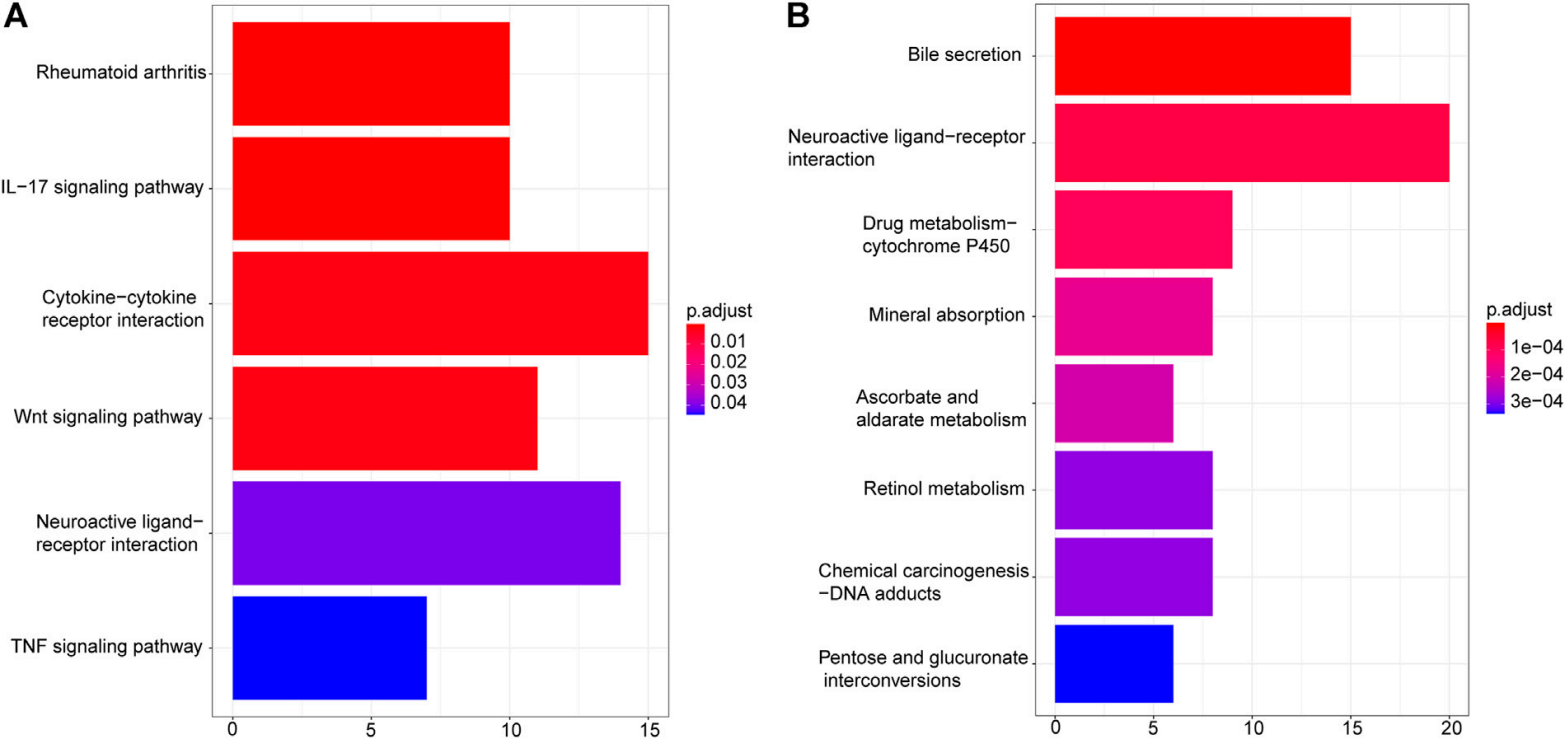
KEGG enrichment analysis of DEGs. **(A)**. Upregulated DEGs. **(B)** Downregulated DEGs.

To understand the possible biological mechanisms that cause the identified changes in the transcriptome data, we conducted the enrichment analysis on the overlapped DEGs using KEGG and GO databases. KEGG pathway enrichment results showed that upregulated DEGs were enriched in “Rheumatoid arthritis”, “IL−17 signaling pathway”, “Cytokine−cytokine receptor interaction”, “Wnt signaling pathway”, “Neuroactive ligand−receptor interaction”, and “TNF signaling pathway” (Figure 3A), while downregulated DEGs were enriched in “Bile secretion”, “Neuroactive ligand−receptor interaction”, “Drug metabolism − cytochrome P450”, “Mineral absorption”, “Ascorbate and aldarate metabolism”, “Retinol metabolism”, “Chemical carcinogenesis − DNA adducts”, and “Pentose and glucuronate interconversions” (Figure 3B).

**FIGURE 4:**
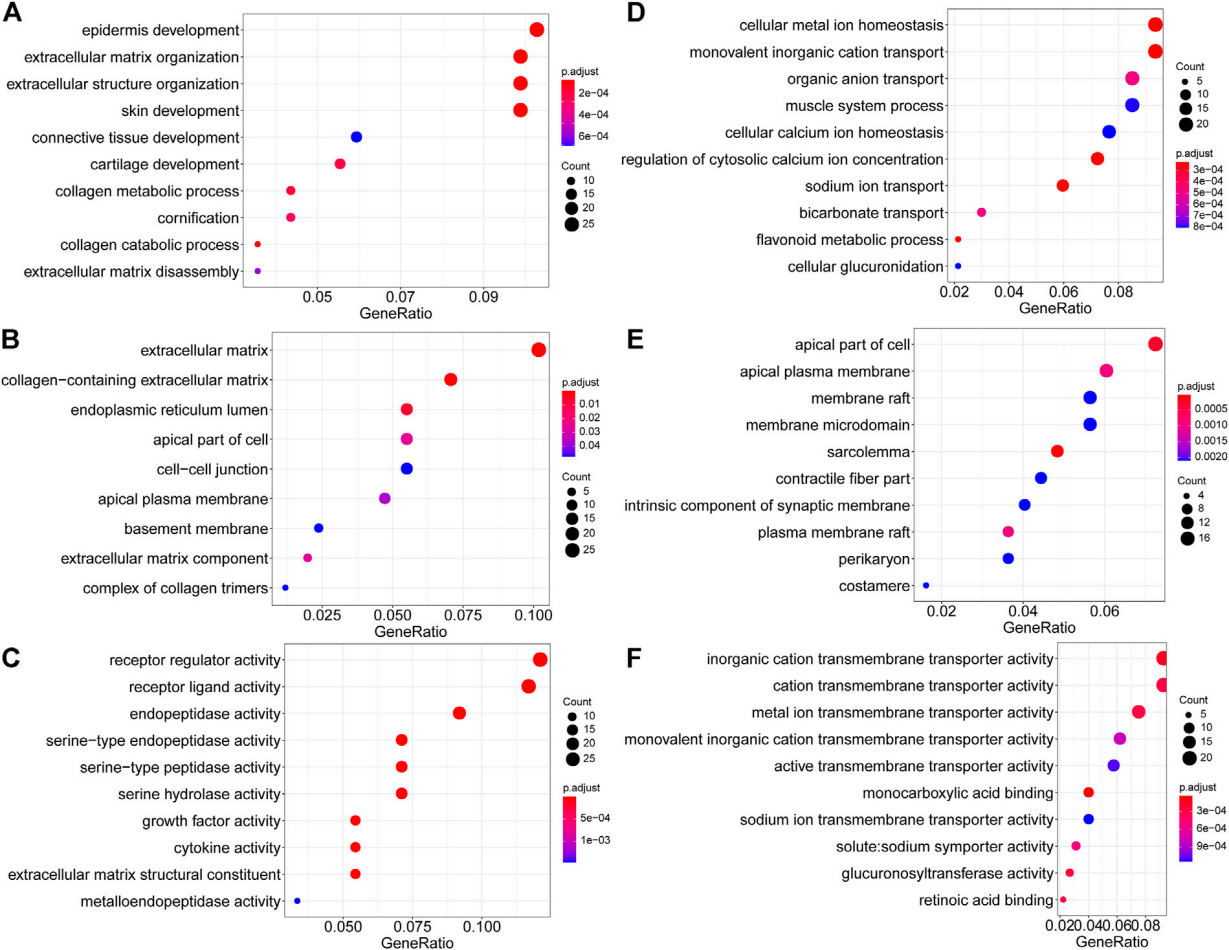
GO enrichment analysis of DEGs. **(A–C)** Upregulated DEGs. **(A)**. Biological process. **(B)** Cellular component. **(C)** Molecular function. **(D–F)**. Downregulated DEGs. **(D)**. Biological process. **(E)** Cellular component. **(F)** Molecular function.

GO terms cover biological process (BP), cellular component (CC), and molecular function (MF). For upregulated DEGs, the enriched BP terms included “epidermis development”, “extracellular matrix organization”, “extracellular structure organization”, “skin development”, “connective tissue development”, “cartilage development”, “collagen metabolic process”, “cornification”, “collagen catabolic process” (Figure 4A). In the CC group, upregulated DEGs were primarily enriched in “extracellular matrix”, “collagen−containing extracellular matrix”, “endoplasmic reticulum lumen”, “apical part of cell”, “cell−cell junction”, “apical plasma membrane”, “basement membrane”, “extracellular matrix component” and “complex of collagen trimers” (Figure 4B). And enriched MF-related terms of upregulated DEGs were “receptor regulator activity”, “receptor ligand activity”, “endopeptidase activity”, “serine−type endopeptidase activity”, “serine−type peptidase activity”, “serine hydrolase activity”, “growth factor activity”, “cytokine activity” and “extracellular matrix structural constituent” (Figure 4C). For downregulated DEGs, the enriched BP terms were “cellular metal ion homeostasis”, “monovalent inorganic cation transport”, “organic anion transport”, “muscle system process”, “cellular calcium ion homeostasis”, “regulation of cytosolic calcium ion concentration”, “sodium ion transport”, “bicarbonate transport”, “flavonoid metabolic process” and “cellular glucuronidation” (Figure 4D). In the CC group, the downregulated DEGs were enriched in “apical part of cell”, “apical plasma membrane”, “membrane raft”, “membrane microdomain”, “sarcolemma”, “contractile fiber part”, “intrinsic component of synaptic membrane”, “plasma membrane raft”, “perikaryon” and “costamere” (Figure 4E). The enriched MF-related terms of the downregulated DEGs were “inorganic cation transmembrane transporter activity”, “cation transmembrane transporter activity”, “metal ion transmembrane transporter activity”, “monovalent inorganic cation transmembrane transporter activity”, “active transmembrane transporter activity”, “monocarboxylic acid binding”, “sodium ion transmembrane transporter activity”, “solute:sodium symporter activity”, “glucuronosyltransferase activity” and “retinoic acid binding” (Figure 4F).

### Hub genes in the PPI network of DEGs

**FIGURE 5:**
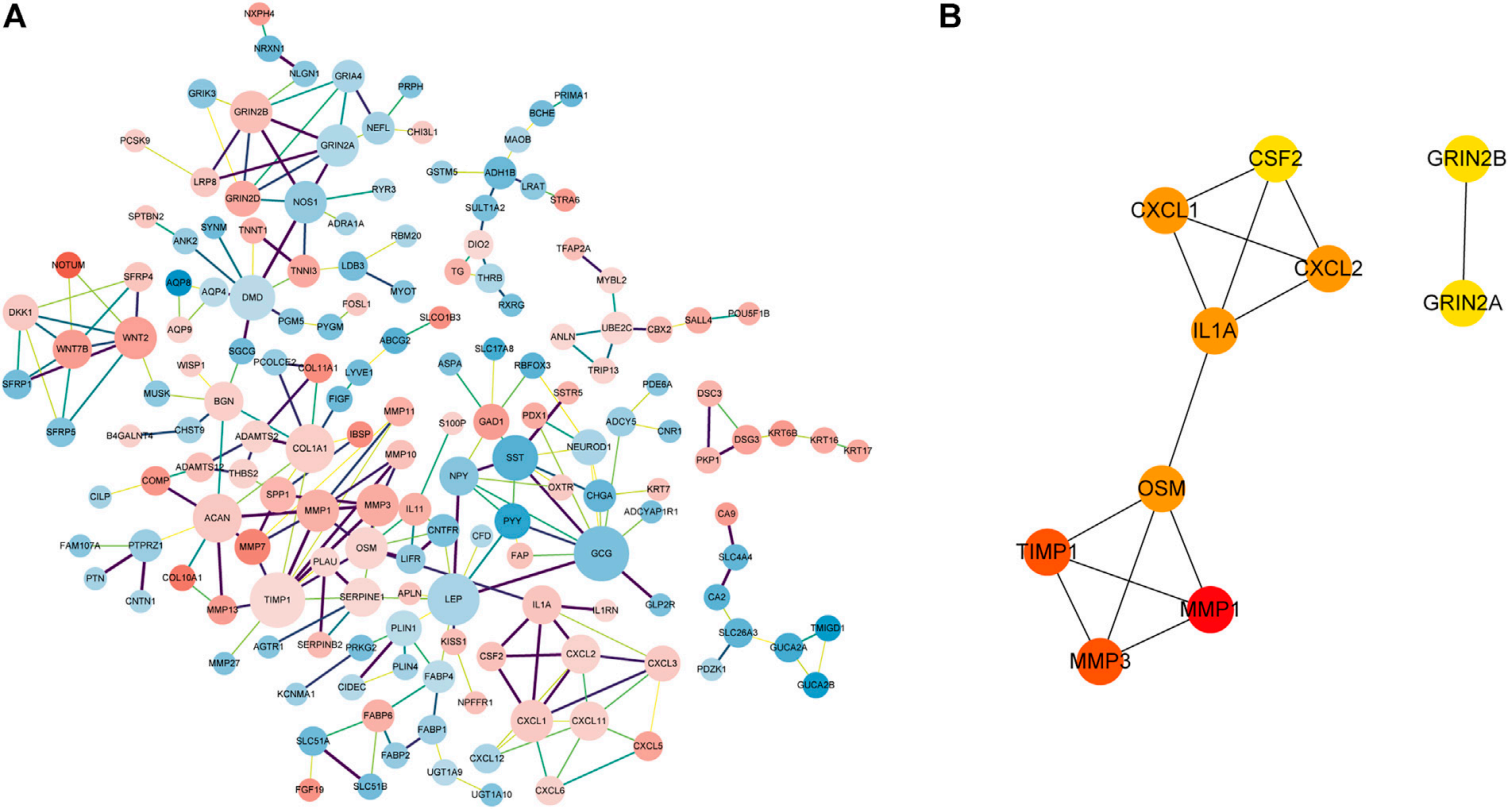
PPI network of DEGs and hub genes in the network. **(A)**. PPI network of DEGs with the interaction score>0.7. The pink nodes indicate significantly upregulated genes and the blue nodes indicate significantly downregulated genes. The edge thickness is proportional to the combined score of the connected genes. The size of the node is proportional to the value of log2|FC|. **(B)** Top 10 hub genes with a higher degree of connectivity.

Based on the STRING online database (http://string-db.org) and Cytoscape software, a PPI network of 164 DEGs and 241 edges was constructed. The minimum required interaction score of each edge were bigger than 0.7 (Figure 5A) which excludes 376 DEGs. The top 10 hub genes according to the node degree were *MMP1, MMP3, TIMP1, OSM, IL1A, CXCL1, CXCL2, CSF2, GRIN2A,* and *GRIN2B* (Figure 5B).

### Correlation between hub genes expression and overall survival

**FIGURE 6:**
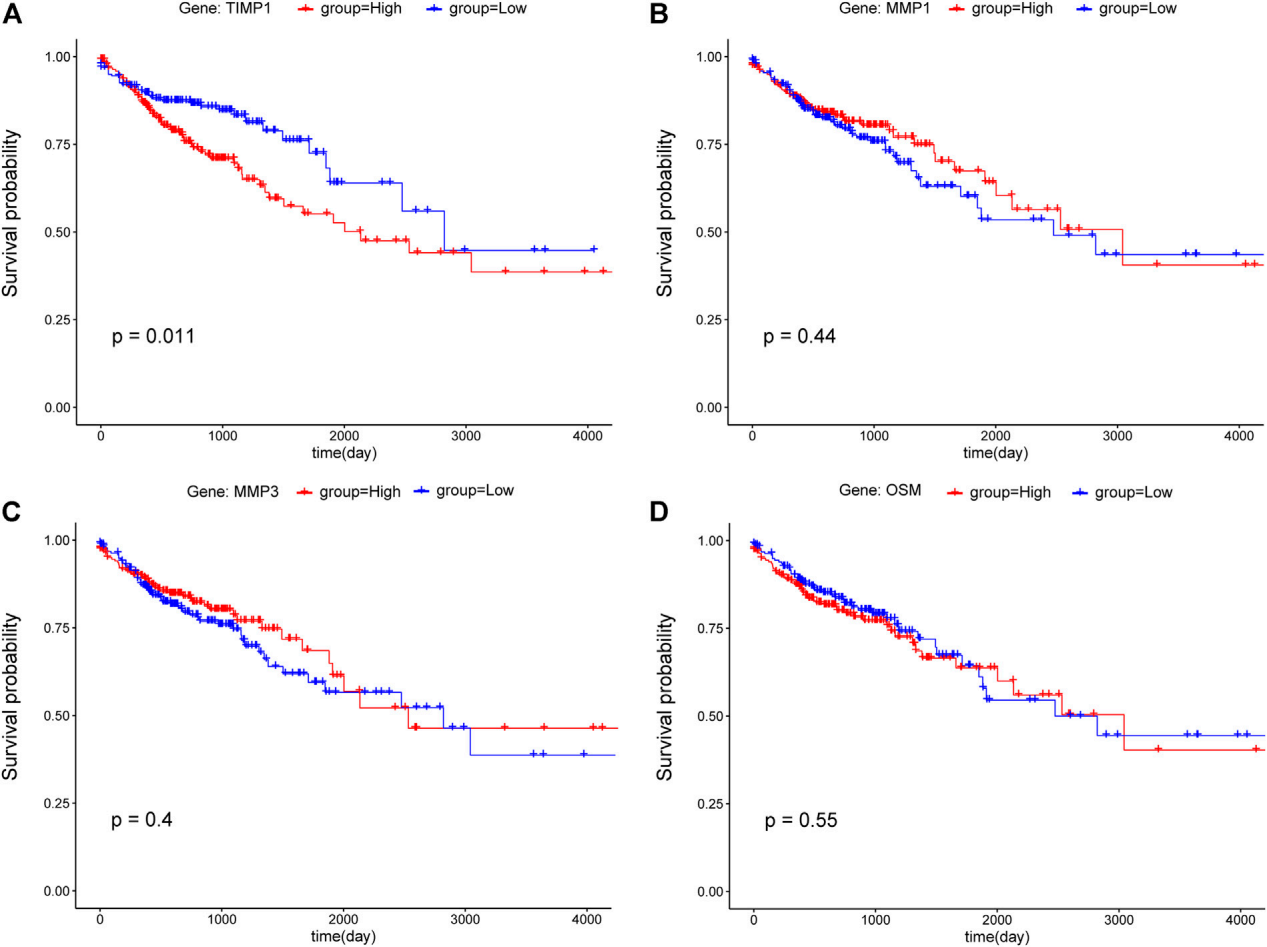
Kaplan-Meier survival curves of CRC patients for the top four hub genes including **(A)** *TIMP1*, **(B)** *MMP1*, **(C)** *MMP3*, and **(D)** *OSM*. According to the median value, gene expression was divided into two groups (red: high; blue: low), and the *p*-value<0.05 was considered statistically significant.

To examine the potential relationship between DEGs and overall survival (OS), a weighted Kaplan Meier survival curves were generated from TCGA data. The survival curves of the top four hub genes were shown in Figure 6, which shown that only *TIMP1* is associated with OS (*p*-value<0.05), and its high expression led to poor prognosis (Figure 6A). Other hub genes are not significantly associated with OS (Figures 6B–D) and Supplementary Figure S1.

### Identification of potential drugs

**FIGURE 7:**
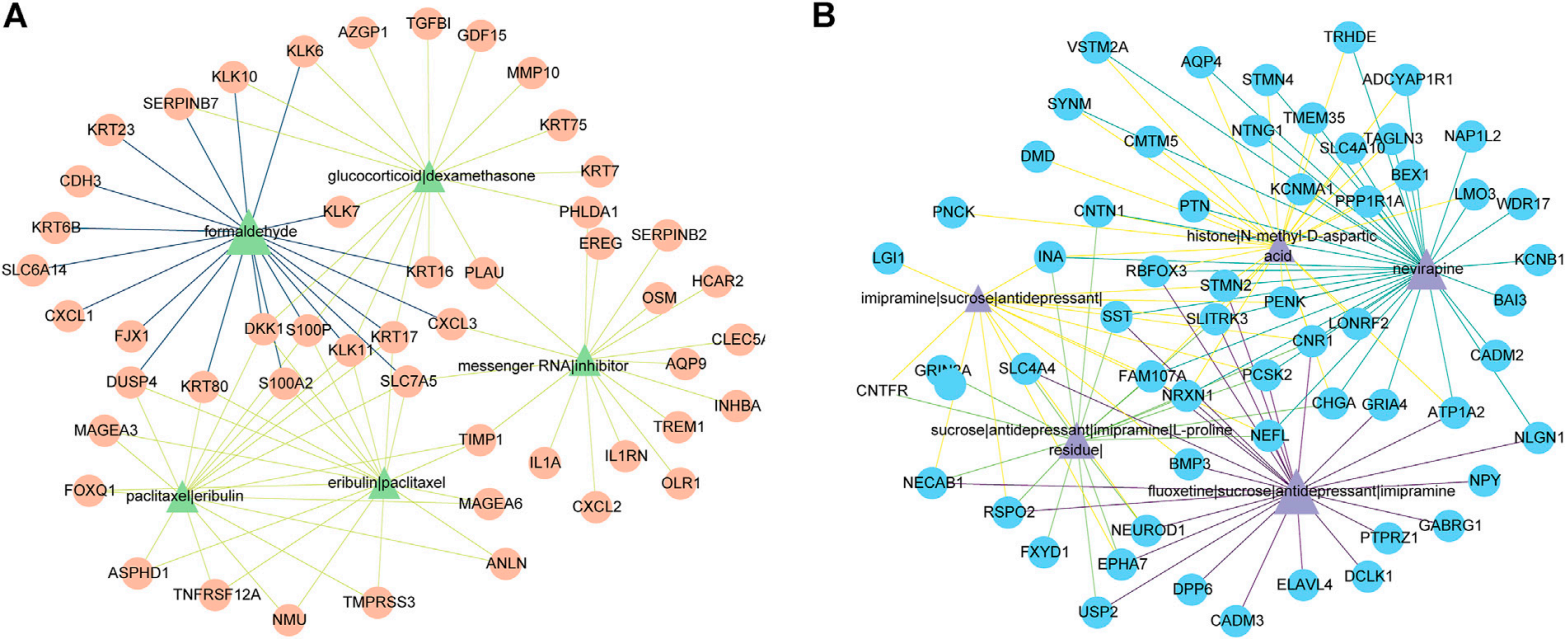
The predicted top five drugs and their gene networks. The color and thickness of the edges are inversely proportional to the *p*-value of drugs and DEGs. **(A)** Five drugs (green triangles) and 50 upregulated DEGs (orange circles). **(B)** Five drugs (purple triangles) and 59 downregulated DEGs (blue circles).

249 potential drugs were predicted according to the DEGs. For example, we plotted five drugs for upregulated DEGs and five drugs for downregulated DEGs in Figure 7. Figure 7 indicated that formaldehyde, glucocorticoid|dexamethasone, paclitaxel|eribulin, messenger RNA|inhibitor, and eribulin|paclitaxel could reverse upregulated DEGs. fluoxetine|sucrose|antidepressant|imipramine, nevirapine, sucrose|antidepressant|imipramine|L-proline residue|, imipramine|sucrose|antidepressant|, and histone|N-methyl-D-aspartic acid could reverse the downregulated DEGs.

Since *TIMP1* is significantly related to the OS of CRC patients, and the high expression of *TIMP1* is correlated to a poor prognosis, we next looked for drugs/small molecules that can reverse the expression of *TIMP1*, which might improve the prognosis of CRC patients. We provided details of the top 10 drugs that can reverse the hub gene *TIMP1* in Table 2, including formaldehyde, paclitaxel|eribulin, erlotinib|dimethyl sulfoxide, glucocorticoid|dexamethasone, antagonist, trichostatin A, rosiglitazone, inhibitor, retinoic acid, and cisplatin. Among them, eight drugs/small molecules were confirmed to be related to *TIMP1* or CRC. It is reported that exposure to formaldehyde can reduce *TIMP1* expression (Kang et al., 2022).

**TABLE 2:** Top 10 drugs for *TIMP1* that were significantly associated with survival rate of CRC patients.

Gene name	Drug/Small molecule	*p*-value	Possible effect	Evidence
*TIMP1*	formaldehyde	1.04403630163397E-06	Formaldehyde is a colorless, irritant, highly active and toxic environmental pollutant, which is used in various industries and products. Inhaled formaldehyde is a human and animal carcinogen that can cause genotoxicity, such as the formation of reactive oxygen species and DNA damage	PMID:35379891
	paclitaxel|eribulin	8.64715E-06	A well-known anticancer agent with a unique mechanism of action. It is considered to be one of the most successful natural anticancer drugs	Unconfirmed
	erlotinib|dimethyl sulfoxide	1.74273E-05	It can interfere with a variety of cellular processes, such as cell proliferation, differentiation, apoptosis and cycle	PMID: 32911099
	glucocorticoid|dexamethasone	3.5055E-05	It has pharmacological effects of anti-inflammatory, anti-endotoxin, inhibiting immunity, anti-shock and enhancing stress response	PMID: 21789017
	antagonist	3.66683E-05	It can bind to receptors and has strong affinity without intrinsic activity (*α* = 0) drugs	Unconfirmed
	trichostatin A	0.0000703775443677834	trichostatin A (TSA), a histone deacetylase (HDAC) inhibitor	PMID: 21520296
	rosiglitazone	0.000141022683961323	Rosiglitazone is a thiazolidinedione insulin sensitizer. Its mechanism of action is similar to that of specific peroxisome proliferator activator *γ* Type a receptor	PMID: 29743857
	Inhibitor	0.000282045	Inhibitors of proteinases or antibodies against certain proteolytic enzymes can prevent tumor invasion and metastasis in experimental conditions	PMID: 23202950
	retinoic acid	0.000564894	Retinoic acid (RA) signal transduction is an important and conservative way to regulate cell proliferation and differentiation. In addition, disturbed RA signaling is associated with the occurrence and progression of cancer	PMID: 34877501
	cisplatin	0.000758387	Cisplatin is an inorganic platinum complex, which can be inhibited by the formation of DNA adducts in tumor cells	PMID: 32329836; PMID: 20607860

## Discussion

In recent decades, CRC, including colon and rectal cancer, has become one of the main causes of cancer-related death around the world (Fuccio et al., 2018; Røed Skårderud et al., 2018; He et al., 2020a; He et al., 2020b). Therefore, it is urgent to find more effective prevention and treatment to reverse this problem (Teer et al., 2017). With the recent progress in the field of medicine and biotechnology, many preclinical and clinical studies have been carried out to reveal the potential mechanism of CRC liver metastasis. Identifying cancer-related marker genes through gene-targeted therapy is a new and effective potentially powerful treatment for CRC (Okugawa et al., 2015; Guo et al., 2017). High throughput sequencing technology provides a new perspective on the genome, transcriptome, and epigenome characteristics of cancer. In this study, we aim to reveal the hub gene of CRC through bioinformatics methods and identify potential drugs or small molecules, to improve the predictive power of CRC and provide a valuable theoretical basis for the clinical treatment of CRC patients.

First, RNA-seq data and clinical information of 478 CRC tumor samples and 41 healthy control samples were downloaded from TCGA. In addition, RNA-seq data of 10 tumor samples and 10 normal samples were obtained from the GSE104836 dataset. Using DESeq2 to detect the DEGs from TCGA and GEO respectively, 2664 DEGs were identified from TCGA, 959 DEGs were identified from the GSE104836 data set, and 540 DEGs appeared in both datasets, including 276 upregulated genes and 264 downregulated genes. KEGG pathway enrichment results showed that upregulated DEGs are enriched in “Rheumatoid arthritis”, “IL−17 signaling pathway”, “Cytokine−cytokine receptor interaction”, “Wnt signaling pathway”, “Neuroactive ligand−receptor interaction”, and “TNF signaling pathway” (Figure 3A). It has been reported that IL − 17 is able to regulate colorectal tumor cells and inhibits their production of cxcl9/10 chemokines, thus prevents the infiltration of CD8 + CTLs and Tregs into CRC tumor, thereby promoting the development of CRC (Chen et al., 2019). Wnt signaling pathway is the key medium of tissue homeostasis and repair. Almost all CRC tumors show overactivation of Wnt pathway (Schatoff et al., 2017; Bian et al., 2020). GO enrichment analysis shows that epidermis development, extracellular matrix, and receptor regulator activity are the most significantly abundant upregulated DEGs in biological processes, cellular components, and molecular function categories. Downregulated DEGs are enriched in “Bile secretion”, “Neuroactive ligand−receptor interaction”, “Drug metabolism−cytochrome P450”, “Mineral absorption”, “Ascorbate and aldarate metabolism”, “Retinol metabolism”, “Chemical carcinogenesis−DNA adducts”, and “Pentose and glucuronate interconversions” (Figure 3B). Previous studies have shown that a high-fat diet promotes the secretion of bile acids, thereby inducing the formation of precancerous lesions and/or aggravating the occurrence of colon tumors (Ocvirk and O'Keefe, 2021). Neuroactive ligand-receptor interactions were associated with other gastrointestinal cancers (Yu et al., 2021). The lack and deficiency of minerals may be related to cancer and increase the risk of cancer; For example, effective absorption of vitamin D can prevent colorectal cancer (Takada and Makishima, 2017).

To identify the key regulating genes in CRC development, a PPI network was constructed based on overlapping DEGs. In this network, edges with association scores <0.7 were filtered out. The PPI network obtained based STRING online database has 164 nodes, and the top 10 hub genes, including *MMP1, MMP3, TIMP1, OSM, IL1A, CXCL1, CXCL2, CSF2, GRIN2A, and GRIN2B*, were identified using Cytoscape. Among them, *TIMP1* is a soluble protein that can be released from endometrial cells, fibroblasts, and cancer cells, which are correlated with the prognosis of various cancers (Peng et al., 2011; Wang et al., 2013). The Kaplan–Meier survival analysis of Zheng et al. showed that *TIMP1* expression was upregulated in CRC tissues and was also connected with poor prognosis in GEPIA datasets (*p*-value = 0.02) (Zheng et al., 2020). Song et al. (2016) reported that *TIMP1* depletion can inhibit the proliferation, migration, and invasion of colon cancer cells, and inhibit the tumorigenesis and metastasis of CRC. Consistent with these studies, our results show that *TIMP1* was up-regulated in CRC samples compared with matched normal tissue samples, and its high expression was associated with poor OS in CRC patients.

Based on DEGs and CREEDS, we made drug predictions for all DEGs (Yang et al., 2020). Previous studies have shown the anti-migration and anti-invasion effects of imipramine, an FDA-approved antidepressant oral drug, on CRC cells (Liu et al., 2016; Alburquerque-González et al., 2020). Fluoxetine has been shown to induce antitumor activity. It was found that fluoxetine could selectively induce concentration-dependent apoptosis in human CRC cells by changing mitochondrial membrane potential and inducing phosphatidylserine translocation to the outer membrane (Marcinkute et al., 2019). In addition, 10 potential drugs were identified to reverse the expression of *TIMP1*. It has been shown that after glucocorticoid treatment, the expression level of *TIMP1* in patients with idiopathic pulmonary fibrosis (IPF) were significantly lower than those before glucocorticoid treatment (*p* < 0.05) (Zhang et al., 2015). Dexamethasone is a synthetic steroid with anti-inflammatory, anti-allergic, and immunosuppressive properties (Sinner, 2019). Trichostatin A is a histone deacetylase (HDAC) inhibitor, which inhibits the growth of CRC cells and induces G1 cell cycle arrest and apoptosis by regulating the downstream target of the JAK2/STAT3 signal (Xiong et al., 2012). A study on the effect of cisplatin on the invasion of ovarian cancer cells showed that the use of cisplatin could reduce the expression of *TIMP1* by 5.0 times (*p* < 0.05) (Karam et al., 2010). It is worth noting that there is no relevant evidence that paclitaxel|eribulin, and Antiagonist are related to the expression of *TIMP1* or the outcome of CRC. Further experiments are needed to verify their effectiveness of action, which may provide a basis for guiding the treatment of CRC patients.

Overall, this study revealed the altered gene expressions and enriched pathways in CRC based on bioinformatics analyses and provides insights for further screening of effective biomolecules for CRC treatment intervention, which is of clinical significance. However, the current research has some limitations. First, because the candidate prognosis-related central DEGs were detected using the data from two independent databases, more datasets were needed to confirm our discoveries. Secondly, experimental methods such as PCR were also needed to verify the DEGs. Third, clinical trials were needed to identify effects of the predicted drugs.

## Conclusion

Our study effectively identified several candidate drug targets through differentially gene expression analysis, hub gene analysis and survival analysis for CRC treatment. We revealed compounds that have the potential to reverse the expressions of the identified DEGs. These findings provide new directions for the diagnosis and treatment of CRC.

## Data Availability

The datasets presented in this study can be found in online repositories. The names of the repository/repositories and accession number(s) can be found in the article/Supplementary Material.
