# Supplementary figures and images for: A bioinformatics framework to identify the biomarkers and potential drugs for the treatment of colorectal cancer
Source: Front Genet. 2022 Sep 27;13:1017539. doi: 10.3389/fgene.2022.1017539 (PMC9551025; doi:10.3389/fgene.2022.1017539)

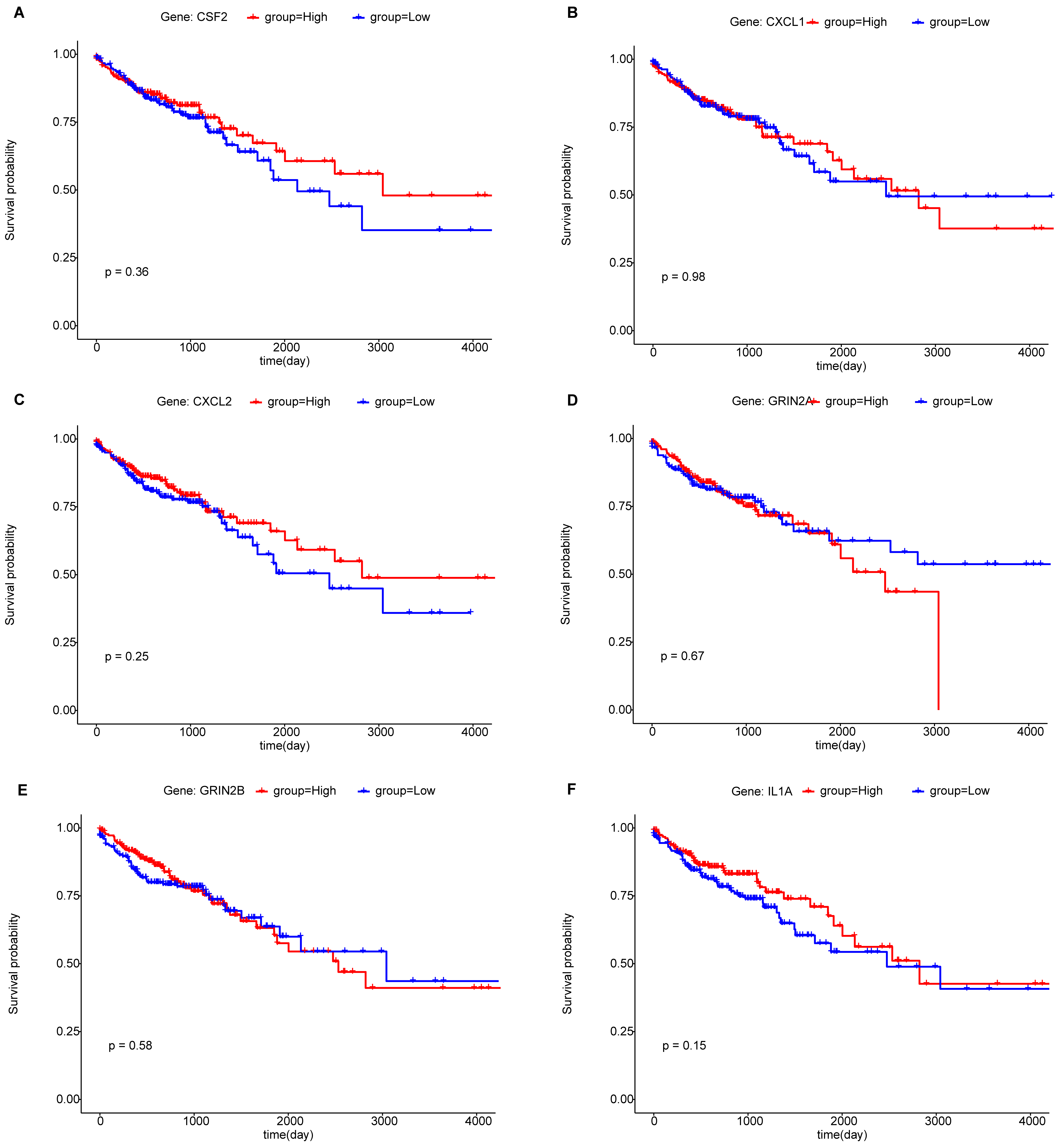

Supplement: Supplementary file 1 [file Image1.TIF]
